# Perceptions of Cardiovascular Health in Underserved Communities

**Published:** 2010-02-15

**Authors:** Lucinda L. Bryant, Nancy P. Chin, I. Diana Fernandez, Lesley A. Cottrell, Joyce M. Duckles, D. Marcela Garces, Thomas C. Keyserling, Carmen D. Samuel-Hodge, Maihan B. Vu, Colleen R. McMilin, Karen E. Peters, Shin-Ping Tu, Annette L. Fitzpatrick

**Affiliations:** Colorado School of Public Health, University of Colorado Denver; University of Rochester School of Medicine and Dentistry, Rochester, New York; University of Rochester School of Medicine and Dentistry, Rochester, New York; West Virginia University, Morgantown, West Virginia; University of Rochester, Rochester, New York; University of Illinois at Rockford, Rockford, Illinois; University of North Carolina at Chapel Hill, Chapel Hill, North Carolina; University of North Carolina at Chapel Hill, Chapel Hill, North Carolina; University of North Carolina at Chapel Hill, Chapel Hill, North Carolina; University of Colorado Denver, Denver, Colorado; University of Illinois at Rockford, Rockford, Illinois, and University of Illinois at Chicago, Chicago, Illinois; University of Washington, Seattle, Washington; University of Washington, Seattle, Washington

## Abstract

**Introduction:**

Cardiovascular disease is the leading cause of deaths and illnesses in US adults, and the prevalence is disproportionately high in underserved populations. In this study, we assessed respondents' understanding of context-specific differences in knowledge and perceptions of disease, risk, and prevention in 6 underserved communities, with the longer-term goal of developing appropriate interventions.

**Methods:**

Thirty-nine small-group sessions and 14 interviews yielded data from 318 adults. Each site's researchers coded, analyzed, and extracted key themes from local data. Investigators from all sites synthesized results and identified common themes and differences.

**Results:**

Themes clustered in 3 areas (barriers to cardiovascular health, constraints related to multiple roles, and suggestions for effective communications and programs). Barriers spanned individual, social and cultural, and environmental levels; women in particular cited multiple roles (eg, competing demands, lack of self-care). Programmatic suggestions included the following: personal, interactive, social context; information in language that people use; activities built around cultural values and interests; and community orientation. In addition, respondents preferred health-related information from trusted groups (eg, AARP), health care providers (but with noticeable differences of opinion), family and friends, and printed materials.

**Conclusion:**

Interventions to decrease barriers to cardiovascular health are needed; these strategies should include family and community context, small groups, interactive methods, culturally sensitive materials, and trusted information sources. New-immigrant communities need culturally and linguistically tailored education before receiving more substantive interventions.

## Introduction

Coronary heart disease (CHD) and stroke are the leading causes of deaths and illnesses in US adults (1,2). More than 80 million American adults live with cardiovascular disease (CVD) (3). Disparities in prevalence in underserved populations are well documented (4-7). For this study, we understand "underserved" to mean social marginalization through the following mechanisms: low-income households and resource-poor communities, racial/ethnic minority status, resource-poor rural areas, limited English proficiency, or recent migration to the United States.

Various ethnic and socioeconomic groups differ in individual health beliefs and practices (8). Knowledge, personal experience, and broader social and cultural influences contribute to perceptions and health beliefs (9). A socioecological perspective (10-13) recognizes that "most public health challenges (eg, encouraging people to exercise regularly, improve their diet, refrain from smoking) are too complex to be understood adequately from single levels of analysis and require more comprehensive approaches that integrate psychological, organizational, cultural, community planning, and regulatory perspectives" (11).

The Prevention Research Centers Cardiovascular Health Intervention Research and Translation Network (PRC CHIRTN) conducted this study to increase understanding of differences in knowledge and perceptions of CVD, risk, and prevention in underserved and understudied populations, with the longer-term goal of addressing the disparities through community-specific interventions and communications. PRC CHIRTN is a collaboration among 6 universities and their partner communities — University of Colorado Denver (UCD), University of Illinois at Chicago (UIC), University of North Carolina at Chapel Hill (UNC), University of Rochester School of Medicine and Dentistry (UR; lead center), University of Washington (UW), and West Virginia University (WVU). Details on its history, mission, and structure are available elsewhere (14). The diversity of the network's partner communities provided a unique opportunity to conduct community-based participatory research to assess barriers to and facilitators of cardiovascular health across populations. In this study, we address issues related to cultural and environmental differences in the knowledge base and perceptions of CVD, risk factors, and prevention, including 1) barriers to and facilitators of cardiovascular health promotion, 2) desired sources of information, and 3) potentially successful avenues for dissemination of information and interventions to reduce the burden of CVD in underserved populations. 

## Methods

### Research procedures

Investigators from all of the 6 PRC CHIRTN sites shared leadership for the project, first to select the research topic and then to reach consensus on a core protocol. Local community teams or advisory committees participated to ensure relevance to partner communities and to assess the language and clarity of discussion questions and research materials for participants. Institutional review boards at each university reviewed and approved the protocols for that site. Investigators met monthly by telephone conference and in March 2008 in person at a national PRC CHIRTN meeting to develop the core protocol and then to track progress and resolve questions.

The protocol included a series of discussion questions for use at all sites and suggested demographic items to document the diversity of samples and allow comparisons between sites. A full list of questions and prompts is available from the corresponding author. Four categories framed the discussion questions:

- knowledge and perceptions of heart disease and stroke
- knowledge and perceptions of prevention
- sources and usefulness of health information
- dissemination methods and strategies

The suggested set of demographic information included age, sex, race/ethnicity, education, geographic location (rural or urban), and length of residence in the United States (if an immigrant population). Several sites also collected information about participants' knowledge of their cardiovascular risk status or history.

Each site chose the appropriate qualitative method for its partner community and site-specific goals, which in some cases extended beyond this study's aims. Five sites (UCD, UIC, UNC, UW, and WVU) conducted small-group sessions of ethnically, geographically, or socioeconomically underserved groups, and 1 (UR) conducted in-depth interviews and neighborhood walking tours with families of urban school children. The facilitators or interviewers for all research sessions were experienced university-based researchers trained in qualitative methods. Following principles of community-based participatory research that promote collaborative partnerships in all phases of research (15), community advisory groups identified and recruited participants representative of each community of interest. On the basis of criteria reviewed and approved by local institutional review boards, participants provided oral or written consent.

Small-group sessions were conducted in English with 2 exceptions; new-immigrant Hispanic sessions were conducted in Spanish (UIC), and Asian immigrant groups were conducted in separate sessions in Mandarin, Cantonese, Vietnamese, or Korean (UW). Small-group sessions took place during the summer of 2007. All sessions were audio-recorded; researchers at each site transcribed (and if necessary translated into English) that site's recordings, removing any personal identifiers. Site-specific details about the groups are provided (Table).

### Analysis

Investigators from all sites assembled a "universal" codebook. The codebook consisted of a priori codes derived from the discussion questions and additional concepts that emerged during analysis. The major coding categories were the following:

- individual factors
- prevention/factors and types
- prevention/being at risk
- disease/symptoms knowledge and understanding
- disease/disease outcome knowledge and understanding
- disease/risk knowledge and understanding
- dissemination
- interventions

Personnel at each site open coded (16) that site's transcripts; at least 2 researchers independently coded and then reconciled differences, using Atlas.ti (Atlas.ti Scientific Software Development, GmbH, Berlin, Germany) software at 4 sites and NVivo/NUD*IST (QSR International, Cambridge, Massachusetts) at 2 sites to facilitate analysis. Investigators then conducted axial coding, combining the original codes into categories by connecting them "in terms of conditions that give rise to them, properties that are common to them, strategies that guide them, and consequences they share" (16). After each site's investigators coded and identified themes, representatives from all sites met face-to-face and then by conference call to identify cross-cutting themes and differences across sites. Investigators then selected representative quotations from their sites to illustrate the research findings.

## Results

### Demographic characteristics

A total of 380 community members participated in qualitative projects across the 6 sites. Participants included 62 children (UR, WVU) with their parents and other interested adults. This analysis includes only the 318 adults (Table). Of the adults, 85% were women (some sites by design sampled only women); 22% were younger than 45 years, 32% were aged 45-64, and 29% were aged 65 or older (additionally, 9% reported age as 35 or older, 1% reported age as 40 or older, and 6% did not provide age information). Slightly more than half (54%) of the adult participants were married.

Recruitment achieved the desired oversampling of at-risk populations. Most of the participants (89%) were of Hispanic ethnicity or nonwhite race; 39% reported themselves as African American, 25% Hispanic, and 24% Asian. More than one-fourth (27%) of respondents had less than a high school education. Although only 2 sites collected information on insurance coverage (UCD, UIC), the rate of those with no insurance (43%) was almost twice the national average (23%) (17). Three sites collected information on health history; 48% of participants self-reported a family history of heart disease (UCD, UIC, UNC), and 28% self-reported a diagnosis of diabetes (UCD, UNC). Rural residents made up 33% of the sample. All small-group sessions at WVU and UCD took place in rural areas (small towns outside of Morgantown, West Virginia, and small towns in the rural San Luis Valley of Colorado), and 51% of UNC respondents identified themselves as rural residents.

### Knowledge of disease, risk factors, disease outcomes, and prevention

Across varied sites and diverse populations, we found that participants who were not recent immigrants had basic general knowledge about CVD, risk factors, disease outcomes, and prevention but experienced locally specific challenges to putting knowledge into practice. Most participants generally recognized high blood pressure and high blood cholesterol levels as risk factors for heart disease, understood the roles of lifestyle and genetics in these risk factors, knew the numbers that indicate high blood pressure and high blood cholesterol levels, and recognized lifestyle and medications as components of reducing risk.

New immigrant populations (UW, UIC) had only rudimentary or incomplete knowledge about CVD, compared with the more established populations. For example, most participants in the UW small-group sessions knew high levels of blood pressure were bad but had difficulties describing what high blood pressure is. Participants in the UIC groups knew that CVD was related to lifestyle factors including diet but did not know the numbers defining high blood pressure and high blood cholesterol levels. Participants in these settings did not understand the importance of taking medication to treat high blood pressure and cholesterol levels.

Participants at all sites had less knowledge about stroke than about heart disease, and they reported fear of stroke. They expressed concern about the loss of independence associated with stroke, the resultant economic implications for families, and the burden on family members who provide care.

### Barriers to putting knowledge into practice

Across small-group sessions and interviews, common socioecological themes emerged as barriers to translating knowledge into healthy behaviors: multiple role demands, lack of economic resources, social and cultural issues that include lack of family and community support for healthy habits, and concerns about health care. Appendix A provides examples in participants' own words.

Particularly for women, their multiple roles as wage-earners, household managers, child care providers, providers of elder care, and people responsible for dealing with health care demands for all family members left them too exhausted to attend to their own health, without time to exercise, and too overextended to prepare nutritious meals at home as often as they liked or knew they should.

Many respondents lamented the high price of fresh fruits and vegetables, the cost of gas for transportation, and the cost of health insurance. Living in resource-poor communities contributed to their difficulties.

Respondents at most sites remarked on the number of fast-food restaurants, citing them as often the only source of meals outside the home. They observed that corner stores with limited fresh foods were more accessible than well-stocked supermarkets. Their neighborhoods often lacked safe walking environments or facilities such as parks, gyms, and playgrounds. Additionally, at some sites, a strong street culture (eg, drugs, violence) competed with families for the attention of their children and created barriers to healthy behaviors in the young. Even for respondents with health insurance, difficult access (eg, lack of transportation) created barriers to appropriate health care. Hispanic respondents noted the lack of providers who speak Spanish, and new immigrants had problems navigating the US health care system and integrating it with traditional beliefs and practices. Asian respondents preferred to use their own community physicians and pharmacists for advice and to rely on their own traditional remedies and products because they perceived that they had access to few other resources for information. Respondents spoke of weather and transportation as barriers to healthy physical activity.

### Facilitators of putting knowledge into practice

Community and social context in some cases facilitated healthy behavior (Appendix B). Family and friends provided knowledge and services such as child care. Learning occurred best in a social context, with information in language that people use and understand. Social support — walking partners, family reinforcement, healthy behavior role models — improved the likelihood of adopting and maintaining healthy changes. On an individual level, several people spoke of the importance and the difficulty of motivation.

### Sources and usefulness of information

Respondents identified a number of information sources that included health care providers, family and friends, printed materials, and other media (eg, Internet, television, radio). Asian immigrants (UW) trust health information from physicians despite communication problems. They also trust information from other providers (eg, acupuncturists), family or friends, and community centers when offered in their native languages. Recent immigrant Hispanic respondents (UIC) reported receiving little CVD information in any form. Nonimmigrant groups (UR, UCD) preferred peer-to-peer discussion groups and family and friends for information and to generate strategies for putting knowledge into practice. Respondents from all sites found currently available print and other media materials of limited usefulness.

### Dissemination strategies

Respondents provided suggestions for disseminating health information into their communities. They identified the following characteristics of successful strategies: responding to the influences of intrapersonal (motivation), interpersonal (social), community (cultural), and institutional contexts. They cited interactive personal contact, use of preferred language ("just being able to present things in a way that people can understand and give them ideas, don't just say — 'here, eat healthy'" [UR]), and tailoring to cultural values and priorities of the local community (eg, promoting community gardens). Respondents recommended family-friendly group sessions ("a little workshop or a little women's retreat or something like . . . , if I experienced it and did some things I would be more likely to incorporate them in my life" [UCD]). They preferred to hear from informed family and friends, especially those who speak of their own experiences; trusted groups (eg, AARP); and health care providers, but not always ("I wouldn't really listen to a doctor. I would listen to a friend that has been through it." [UNC]). Asian and Hispanic immigrants (UIC, UW) expressed the need for educational materials in their own languages and for dissemination approaches sensitive to community and culture.

## Discussion

The diversity of PRC CHIRTN communities and successful sampling made it possible to collect new insights into knowledge, perceptions, and preferred dissemination methods that will facilitate community-specific prevention activities. The results from this study indicate that many people (but not new immigrants) have adequate knowledge of heart disease and its risk factors, including information about healthy lifestyles, but are less well informed about stroke. Another study found that underserved people with known elevated risk of CVD (18) had limited risk-factor knowledge, although women, rural residents, and those with higher incomes had more awareness and knowledge. The predominance of women in our study may account for some of the difference. New immigrants — Hispanic and Asian — have a more immediate need for basic information.

Even with adequate knowledge, members of underserved populations have difficulty putting what they know into practice. In particular, they, especially women, identify barriers related to multiple family- and work-based responsibilities (8,19-23) and a need for strategies and programs to promote and facilitate self-care. As previously reported in underserved groups, other barriers include economic constraints (19,21,22), social and cultural concerns (24), and access (22).

Participants offered valuable suggestions for culturally appropriate, community-specific approaches to promote cardiovascular health:

- Interactive, hands-on programs in small groups, including social support to bolster motivation to comply with prevention and treatment regimens.
- Information from informed peers who have personal experience to relate. We know from the literature and experience in our communities that lay health workers, who have an intimate understanding of their community's sociocultural background, experiences, challenges, and strengths are in a unique position to provide peer support for community members (10,25,26).
- "Real-world" vocabulary in the preferred language. A need exists particularly in low-literacy communities for accurate, credible, and current information (27) in clear, conversational language (28), but research to date finds only mixed results for interventions to overcome literacy barriers (29). One of the PRC CHIRTN sites (UW) has had success using photography to solicit information for messaging on cardiovascular health topics, especially among older Asian immigrants (30), and 2 sites (UW, UIC) have developed audio novellas to increase levels of cardiovascular health information in new-immigrant Hispanic groups.
- Improved communication with and information from health care providers.
- Assistance with child care and transportation.
- More community resources for physical activity and healthy food.

These suggestions emphasize the need to engage the community and the consumer in assessing needs and developing materials and programs, as recommended by the National Expert Panel on Community Health Promotion (31). Although most study participants in this study reported basic CVD knowledge, many lacked resources or motivation to apply it. Effective interventions will need to address daily competing priorities and barriers to improving healthy behaviors. Programs that focus on problem solving (a cognitive strategy) (32,33) or motivational interviewing (a behavioral strategy) (34) offer promise. As an example, responding to women in this study, 3 PRC CHIRTN sites (UCD, UNC, WVU) conducted a second round of small-group sessions to tailor a problem-solving intervention to the community context, with the goal of improving participants' capacity to manage their situations.

### Limitations and strengths

Small-group sessions and interviews may not adequately mirror the characteristics of the community or population that they represent. The diversity of settings we reached suggests that the results described here reflect similarities and differences across underserved groups with increased CVD risk. Although the research sites used different data collection methods, the development of common protocols, discussion questions, and coding schemes means that we spoke of the same issues in the same ways. Investigators from all sites collaborated to conduct the highest level of analysis and synthesis, which increased the rigor of the results and the implications drawn from them.

### Implications

Public health practitioners and programs must reach people at high risk and engage them in prevention activities. The results of this study make it clear that we need to move beyond individual- and knowledge-based interventions to new approaches that involve social marketing; environmental change to improve access to nutritious food, physical activity, and health care; and strategies tailored to the context.

This study identified a number of common barriers to cardiovascular health across groups of underserved populations that community interventions may address. Although sensitivity to unique cultural settings must be considered, many similarities exist across groups concerning suggestions for approaches to improve knowledge and CVD prevention practice. The socioecological perspective provides a framework for creating multifaceted disease prevention interventions and related communication strategies that simultaneously target the different levels of influence and build on community strengths.

## Figures and Tables

**Table.** Description of Methods and Participants by Site, Prevention Research Centers Cardiovascular Health Intervention Research and Translation Network, 2007

**Characteristic**	**Location of the Study**					

	**University of Colorado Denver^a^**	**University of Illinois at Chicago^b^**	**University of North Carolina at Chapel Hill^c^**	**University of Rochester^d^**	**University of Washington^e^**	**West Virginia University^f^**
**Methods**	4 small-group sessions	8 small-group sessions	10 small-group sessions	14 in-home interviews	8 small-group sessions	9 small-group sessions
**No. of participants**	18	64	115	14	77	30
**Female, %**	100	73	100	86	64	93
**Age range, y**	35-65	≥18	40-85	25-57	≥35	35-65
**Education, %**						
Less than high school graduate	11	41	9	0	27	Data not collected
High school graduate or some college	50	53	66	64	66	Data not collected
College graduate	39	6	25	36	34	Data not collected
**Married, % **	72	63	44	21	81	Data not collected

a Participants were primarily rural Hispanic women (72% Hispanic, 28% non-Hispanic white).

b Participants were rural Hispanic immigrants (100%).

c Participants were urban and rural African American women (100%).

d Participants were urban low-income families with elementary school-aged children (14% Hispanic, 14% non-Hispanic white, and 71% African American).

e Participants were Asian American adult immigrants (33% spoke Mandarin, 23% Korean, 22% Cantonese, and 22% Vietnamese).

f Participants were rural adults and children (96.7% non-Hispanic white).

## Appendices

### Appendix A. Barriers to and Constraints on Behaviors for Cardiovascular Health: Key Themes and Representative Quotations

**Multiple role demands **

**(mentioned in University of Colorado Denver [UCD], University of North Carolina at Chapel Hill [UNC], University of Illinois at Chicago [UIC], University of Rochester [UR], University of Washington [UW], and West Virginia University [WVU])**

"Sad. I'm so motivated for everything else but not for something that would benefit me . . . I think I tend to take care of other people, get the chores done, and make sure that the food is pleasing, and you know that everyone else is taken care of before I think about what I need right now." (UCD)

"We make sure kids get their care but for whatever reason, mom just won't take the time out [for herself]." (UNC)

"I don't have enough time to make a healthy dinner or to eat a balanced meal." (UIC)

"I wasn't paying attention at the time [to my weight], I'm too busy working, or going to school or whatever it is, not getting enough exercise, and sometimes I think at least in our family because of obesity is so rampant I think that even though we're eating well, not getting enough exercise does as much damage." (UR)

**Economic resources (mentioned in UCD, UIC, and UR)**

One woman who had been a vegetarian in Los Angeles found Rochester food prices too high to continue to eat that way. A respondent who lost his business and with it his insurance was reluctant to pay out of pocket for blood pressure treatment and reserved medical visits to emergency situations. (UR)

The rural immigrant respondents had trouble finding day care for their children. (UIC)

"Sometimes I don't have enough money to buy healthier food and I have to eat whatever I have." (UIC)

"I've been self-employed and really haven't been able to afford health insurance, so we are definitely lacking going to the doctor and having checkups and physicals and we are not going to a doctor unless something happens." (UR)

"A fear of not knowing what I'm going to pay when I go in [for health care]." (UCD)

**Social, cultural, and environmental issues (mentioned in UCD, UIC, UR, UW, WVU, and UNC)**

"We as Hispanics like greasy food . . . because people are not educated on the way we should eat and generally Hispanics don't realize that we suffer a disease until the last moment." (UIC)

"I've become so lazy now since living here. It is because the car takes you everywhere, you don't have to walk." (UW)

"That's all we have. That's the choices that are given to us. So our population is fast food. We've been raised on that mentality. That's what we're supposed to do. You're supposed to take your kids to McDonald's." (WVU)

"Diet is related to heart disease. You know, especially the black community [diet] because of the way we eat. Fried foods to fatty foods. Everything we eat." (UNC)

"Oh, don't talk about McDonald's. All the corner stores they are selling junk food. You know there is a grocery store and I didn't even know it, selling 'hamburgers' and the ready-made hamburgers in a package. And they buy it . . . and the corner store has a microwave and they just warm it up and they eat it." (UR)

"I guess I question whether or not they have gotten the information. I suspect that they have gotten the information and there's just the way their lives are; it's there isn't time or there isn't money, or something makes it not easy to do." (UCD)

"Whom should I trust? I am very confused . . . I don't know whom to listen to. I don't know how to read English and there is nothing in my language. . ." (UW)

**Concerns about health care (mentioned in UCD, UIC, UNC, UR, and UW)**

"Or maybe what I feel like when I go to the doctors is that they don't have enough time to sit and talk with you." (UCD)

Culture-specific remedies without evidence: "Sometimes it helps to bring down the blood pressure if you drink Chinese herbal tea"; "I know celery and cilantro also helps"; "Zhu Zi Long . . . is a kind of herbal medicine . . . [I take] just occasionally when I feel 'heat' inside my body." (UW)

"Lack of medical professionals who speak Spanish." (UIC)

"I think a lot of people stay away from the doctor because they aren't taken seriously . . . they [doctors] just don't think it is a big deal when you tell them you hurt. You feel like you are not important as a patient." (UNC)

"We came here not long ago. Always we see differences between American doctors and doctors in China. For us, we don't know if it is a special obstacle. Every time I went to the hospital, the doctor told me to go home. But I was not well and I felt uncomfortable. So I checked the Chinese Web site and went to Chinese drug store to buy Chinese medicine and I took it. Then I felt fine." (UW)

"And then another thing here in the valley is we lose our doctors monthly." (UCD)

Note: These are selected quotations; absence of a quotation from a site does not mean that the topic was not mentioned there.

### Appendix B. Facilitators of Behaviors for Cardiovascular Health: Key Themes and Representative Quotations

**Friends, families, and social support (UCD, UNC, and WVU)**

"I've learned a lot from my friends and family." (UCD)

"I used to have some walking partners and we would call each other at 5 in the morning and go walking down at the community center. One of them couldn't walk as much as the rest of us but we would all walk together. That kept my weight down." (UNC)

"If my family wants me to work on these things, I'll do it but I need a lot of reinforcement." (WVU)

"[In a family] everybody eats the same thing, everybody helps each other out." (UNC)

"It would be nice to have a support group that you go and say how we've done . . . we're like little kids, we need to be rewarded to keep us on track." (UCD)

**Social environment or context (including school and work sites; community access to healthy foods, health care) (UCD, UR, and UW)**

"Sometimes it's not that easy with strong personal will. So it's important to get lectures and instructions from some kind of program and meetings." (UW)

"It is good to get support from friends with good lifestyle." (UW)

"I think we started eating a little more healthy, we're slowly putting some healthy choices in there. I did lose 12 pounds on the program [responding to a question about a worksite program]." (UR)

"I think we need to look at our communities too." (UCD)

**Personal motivation (UCD, UIC,** **UNC** **, and UW)**

"When you go to the grocery stores, you know, you have to change your buying habits. Stop buying, you know how you buy a lot of junk foods, I quit buying junk food. The ice cream. When I'd like ice cream, I started buying the sorbet or the low-fat ice creams. When you're shopping, you know the things that you really like to eat, you can't go down the aisle . . . and not putting the foods in your cabinet that will be trouble. If they're not there, I won't worry about it." (UNC)

"A huge clue is you have to be motivated to do it." (UCD)

"You can control time. All those bad eating habits we should dominate them." (UIC)

Note: These are selected quotations; absence of a quotation from a site does not mean that the topic was not mentioned there.

## References

[1] (2006). Health United States 2006.

[2] (2006). Addressing the nation's leading killers, 2006.

[3] Rosamond W, Flegal K, Furie K, Go A, Greenlund K, Haase N (2008). Heart disease and stroke statistics: 2008 update: a report from the American Heart Association Statistics Committee and Stroke Statistics Subcommittee. Circulation.

[4] Kurian AK, Cardarrelli KM (2007). Racial and ethnic differences in cardiovascular disease risk factors: a systematic review. Ethn Dis.

[5] Kanjilal S, Gregg EW, Cheng YJ, Zhang P, Neldon DE, Mensah G (2006). Socioeconomic status and trends in disparities in 4 major risk factors for cardiovascular disease among US adults, 1971-2002. Arch Intern Med.

[6] Mensah GA, Mokdad AH, Ford ES, Greenlund KJ, Croft JB (2005). State of disparities in cardiovascular health in the United States. Circulation.

[7] Colleran KM, Richards A, Shafer K (2007). Disparities in cardiovascular disease risk and treatment: demographic comparison. J Investig Med.

[8] Samuel-Hodge CD, Headen SW, Skelly AH, Ingram AF, Keyserling TC, Jackson EJ (2000). Influences on day-to-day management of type 2 diabetes among African-American women: spirituality, the multi-caregiver role, and other social context factors. Diabetes Care.

[9] Cristancho S, Garces DM, Peters K, Mueller B (2008). Listening to rural Hispanic communities in the Midwest: a community-based participatory assessment of major barriers to health care access and use. Qualitative Health Research.

[10] DeBate R, Plescia M, Joyner D, Spann L (2004). A qualitative assessment of Charlotte REACH: an ecological perspective for decreasing CVD and diabetes among African Americans. Ethn Dis.

[11] Stokols D (1996). Translating social ecological theory into guidelines for community health promotion. Am J Health Promot.

[12] Sallis JF, Owen N, Glanz K, Rimer BK, Lewis FM (2004). Ecological models of health behavior. Health behavior and health education: theory, research, and practice.

[13] Weisner TS, Weisner TS (2005). Introduction. Discovering successful pathways in children's development: mixed methods in the study of childhood and family life.

[14] Farris RP, Pearson T, Fogg T, Bryant L, Peters K, Keyserling T (2008). Building capacity for heart disease and stroke prevention research: the Cardiovascular Health Intervention Research and Translation Network. Health Promot Pract.

[15] Israel BA, Schulz AJ, Parker EA, Becker AB (1998). Review of community-based research: assessing partnership approaches to improve public health. Ann Rev Public Health.

[16] Strauss A, Corbin J (1990). Basics of qualitative research: grounded theory procedures and techniques.

[17] Cohen RA, Martinez ME (2007). Health insurance coverage: early release of estimates from the National Health Interview Survey, January – June 2007.

[18] Homko CJ, Santamore WP, Zamora L, Shirk G, Gaughan J, Cross R (2008). Cardiovascular disease knowledge and risk perception among underserved individuals at increased risk of cardiovascular disease. J Cardiovasc Nurs.

[19] Brett JA, Heimendinger J, Boender C, Morin C (2002). Using ethnography to improve intervention design. Am J Health Promot.

[20] Gatewood JG, Litchfield RE, Ryan SJ, Myers JD, Geadelmann JD, Pendergast JF (2008). Perceived barriers to community-based health promotion program participation. Am J Health Behav.

[21] Punzalan C, Paxton KC, Guentzel H, Bluthenthal RN, Staunton AD, Mejia G (2006). Seeking community input to improve implementation of a lifestyle modification program. Ethn Dis.

[22] Speck BJ, Hines-Martin V, Stetson BA, Looney SW (2007). An environmental intervention aimed at increasing physical activity levels in low-income women. J Cardiovasc Nurs.

[23] Goldman RE, Parker DR, Eaton CB, Borkan JM, Grambling R, Cover RT (2006). Patients' perceptions of cholesterol, CVD risk, and risk communication strategies. Ann Fam Med.

[24] Harralson TL, Cousler Emig, Polansky M, Walker RE, Otero Cruz, Garcia-Leeds C (2007). Un corazon saludable: factors influencing outcomes of an exercise program designed to impact cardiac and metabolic risks among urban Latinas. J Community Health.

[25] Kim S, Koniak-Griffin D, Flaskerud JH, Guarnero PA (2004). The impact of lay health advisors on cardiovascular health promotion using a community-based participatory approach. J Cardiovasc Nurs.

[26] Boutin-Foster C, George KS, Samuel T, Fraser-White M, Brown H (2008). Training community health workers to be advocates for health promotion: efforts taken by a community-based organization to reduce health disparities in cardiovascular disease. J Community Health.

[27] Institute of Medicine (2004). Health literacy: a prescription to end confusion.

[28] Merriman B, Ades T, Seffrin JR (2002). Health literacy in the information age: communicating cancer information to patients and families. CA Cancer J Clin.

[29] Pignone M, DeWalt DA, Sheridan S, Berkman N, Lohr KN (2005). Interventions to improve health outcomes for patients with low literacy: a systematic review. J Gen Intern Med.

[30] Fitzpatrick AL, Steinman LE, Tu S-P, Ly KA, Ton TGN, Yip M-P (2009). Communicating with pictures: perceptions of cardiovascular health in Asian immigrants. Am J Public Health.

[31] Navarro AM, Voetsch KP, Liburd LC, Giles HW, Collins JL (2007). Charting the Future of Community Health Promotion: Recommendations From the National Expert Panel on Community Health Promotion. Prev Chronic Dis.

[32] Hill-Briggs R, Gemmell L (2007). Problem solving in diabetes self-management and control: a systematic review of the literature. Diabetes Educ.

[33] D'Zurilla TJ, Nezu AM, Maydeu-Olivares A (2002). Social problem-solving inventory-revised (SPSI-R) manual.

[34] Rollnick S, Mason P, Butler C (1999). Health behavior change: a guide for practitioners.

